# Supplementary material for: A Likelihood Approach for Real-Time Calibration of Stochastic Compartmental Epidemic Models
Source: PLoS Comput Biol. 2017 Jan 17;13(1):e1005257. doi: 10.1371/journal.pcbi.1005257 (PMC5240920; doi:10.1371/journal.pcbi.1005257)
Supplement: S1 File — (TAR.GZ) [file pcbi.1005257.s014.tar.gz › HSPH_Online-SI-Revision/MSS10/n100-severe/n100-severe_NEW2_table-8.pdf]

1	newly 3 weeks spec	2276.	2339.91	{1605., 3062.}	19.7632	0.0881921	newly 3 weeks cumul	4764.	4983.79	{3513., 6370.}	19.9574	0.0869124	newly 5 weeks spec	4385.	3952.09	{2881., 4922.}	15.5979	0.0784749	newly 5 weeks cumul	12 438.	12 156.1	{8511., 14 958.}	16.3556	0.0766974	newly 58 weeks cumul	64 638.	36 214.	{31 513., 39 645.}	43.9742	0.255445
2	newly 3 weeks spec	951.	3496.51	{2008., 6159.}	267.667	0.522138	newly 3 weeks cumul	1661.	5006.61	{2849., 8434.}	201.868	0.442317	newly 5 weeks spec	4438.	14 424.7	{10 241., 19 809.}	225.027	0.496919	newly 5 weeks cumul	8385.	27 947.3	{17 197., 43 538.}	233.301	0.499327	newly 65 weeks cumul	54 065.	82 657.1	{75 520., 91 685.}	52.8847	0.182352
3	newly 3 weeks spec	788.	1693.03	{344., 3284.}	137.897	0.370272	newly 3 weeks cumul	1184.	2354.11	{603., 4574.}	120.205	0.33037	newly 5 weeks spec	4947.	9211.04	{2023., 17 699.}	113.474	0.37967	newly 5 weeks cumul	8233.	16 226.6	{3532., 31 092.}	122.659	0.36907	newly 66 weeks cumul	68 234.	74 653.1	{56 046., 92 794.}	20.1131	0.122306
4	newly 3 weeks spec	1410.	5541.	{3209., 9967.}	293.797	0.549747	newly 3 weeks cumul	2503.	8239.27	{4982., 14 306.}	230.266	0.478211	newly 5 weeks spec	4326.	14 898.	{10 666., 18 159.}	244.383	0.528126	newly 5 weeks cumul	9460.	34 182.2	{23 470., 48 257.}	261.334	0.540083	newly 64 weeks cumul	66 493.	77 398.7	{60 410., 92 161.}	20.3049	0.079078
5	newly 3 weeks spec	2748.	2596.49	{1988., 3170.}	14.3162	0.0668543	newly 3 weeks cumul	5863.	5716.55	{4305., 7000.}	13.3858	0.0607446	newly 5 weeks spec	4410.	3990.32	{3183., 4667.}	12.858	0.0622562	newly 5 weeks cumul	13 943.	13 092.6	{10 104., 15 784.}	12.2984	0.0576845	newly 58 weeks cumul	66 068.	35 152.7	{30 980., 38 916.}	46.7932	0.275991
6	newly 3 weeks spec	1016.	916.86	{105., 1707.}	52.6811	0.438463	newly 3 weeks cumul	1815.	1599.54	{215., 2957.}	47.2187	0.350703	newly 5 weeks spec	3575.	2968.97	{240., 5787.}	54.2979	0.51403	newly 5 weeks cumul	7380.	6320.03	{803., 11 681.}	52.4031	0.416282	newly 64 weeks cumul	66 876.	40 995.9	{32 658., 56 507.}	40.119	0.36242
7	newly 3 weeks spec	815.	1677.04	{752., 2566.}	112.189	0.246973	newly 3 weeks cumul	1311.	2436.17	{1150., 3624.}	92.6583	0.219239	newly 5 weeks spec	3565.	7940.59	{3786., 15 256.}	126.236	0.31245	newly 5 weeks cumul	6614.	14 428.3	{6861., 25 718.}	122.287	0.294908	newly 65 weeks cumul	69 295.	83 714.9	{70 741., 93 559.}	21.5511	0.0834883
8	newly 3 weeks spec	1680.	2357.56	{2124., 2577.}	40.331	0.1458	newly 3 weeks cumul	3165.	4443.06	{4014., 4866.}	40.381	0.146146	newly 5 weeks spec	3946.	5873.17	{5481., 6361.}	48.8386	0.171931	newly 5 weeks cumul	9677.	14 196.6	{13 111., 15 491.}	46.7044	0.165512	newly 61 weeks cumul	59 021.	89 497.9	{87 771., 92 297.}	51.6375	0.180702
9	newly 3 weeks spec	240.	663.44	{2., 538.}	281.683	0.842684	newly 3 weeks cumul	369.	918.87	{32., 841.}	240.675	0.581773	newly 5 weeks spec	1645.	1138.68	{0., 2657.}	102.213	1.32566	newly 5 weeks cumul	2651.	3086.11	{38., 4501.}	139.395	0.888379	newly 66 weeks cumul	62 553.	36 417.1	{38., 70 475.}	48.4096	0.810788
10	newly 3 weeks spec	1249.	680.4	{243., 1057.}	48.2818	0.384465	newly 3 weeks cumul	2193.	1355.01	{540., 2221.}	42.389	0.307304	newly 5 weeks spec	4136.	1607.36	{404., 2761.}	61.1373	0.530698	newly 5 weeks cumul	8808.	4028.9	{1356., 6450.}	54.6424	0.439764	newly 59 weeks cumul	62 587.	35 165.2	{31 920., 40 943.}	43.8138	0.295639
11	newly 3 weeks spec	804.	9641.83	{1177., 21 359.}	1107.94	1.024	newly 3 weeks cumul	1294.	14 341.1	{1529., 26 584.}	1017.7	0.94852	newly 5 weeks spec	3948.	10 968.1	{4668., 15 222.}	186.586	0.534111	newly 5 weeks cumul	7140.	38 173.3	{16 903., 51 156.}	440.675	0.763209	newly 65 weeks cumul	63 055.	50 969.1	{49 028., 52 031.}	20.7572	0.173467
12	newly 3 weeks spec	2111.	2103.77	{1482., 2810.}	20.4477	0.0923257	newly 3 weeks cumul	4254.	4322.42	{2992., 5517.}	19.2134	0.0861469	newly 5 weeks spec	4443.	3848.95	{2785., 4855.}	19.194	0.0973801	newly 5 weeks cumul	11 913.	11 162.4	{7959., 14 113.}	17.811	0.0854992	newly 58 weeks cumul	66 884.	34 550.3	{31 560., 36 801.}	48.343	0.287629
13	newly 3 weeks spec	2345.	2174.13	{918., 3364.}	33.9672	0.184885	newly 3 weeks cumul	5116.	4727.55	{2291., 6937.}	29.7484	0.163033	newly 5 weeks spec	4572.	3383.45	{1073., 5309.}	32.946	0.228598	newly 5 weeks cumul	13 208.	10 984.4	{5256., 16 628.}	30.8116	0.187532	newly 58 weeks cumul	66 392.	29 343.5	{11 501., 35 852.}	55.8027	0.389176
14	newly 3 weeks spec	1956.	2147.76	{1171., 2964.}	30.4611	0.128871	newly 3 weeks cumul	4187.	4596.76	{2662., 6366.}	28.5269	0.119612	newly 5 weeks spec	3466.	3569.49	{2404., 4870.}	22.0782	0.105716	newly 5 weeks cumul	10 276.	11 085.	{6649., 14 974.}	26.1322	0.113476	newly 58 weeks cumul	61 739.	32 138.5	{31 026., 35 354.}	47.9446	0.290887
15	newly 3 weeks spec	1396.	986.13	{339., 1535.}	39.9191	0.26382	newly 3 weeks cumul	2586.	1955.66	{778., 2955.}	36.1694	0.226591	newly 5 weeks spec	3233.	2228.1	{775., 3695.}	40.3576	0.267358	newly 5 weeks cumul	7869.	5706.49	{2335., 8903.}	38.3961	0.248022	newly 60 weeks cumul	52 704.	39 422.3	{32 405., 47 442.}	27.6089	0.147204
16	newly 3 weeks spec	1498.	1613.86	{921., 2146.}	28.4379	0.123643	newly 3 weeks cumul	2968.	3259.3	{1993., 4170.}	28.308	0.119159	newly 5 weeks spec	3246.	3262.23	{1812., 4102.}	23.4858	0.114293	newly 5 weeks cumul	8486.	8911.33	{5268., 11 881.}	25.3654	0.114717	newly 59 weeks cumul	63 449.	48 606.	{32 623., 84 356.}	41.8137	0.225599
17	newly 3 weeks spec	711.	2271.79	{472., 5459.}	234.468	0.414556	newly 3 weeks cumul	1220.	3358.08	{842., 7812.}	189.634	0.373106	newly 5 weeks spec	2605.	7190.82	{1761., 15 472.}	189.864	0.429542	newly 5 weeks cumul	5099.	15 293.8	{3613., 37 338.}	213.548	0.435339	newly 65 weeks cumul	53 195.	59 623.4	{38 306., 70 987.}	19.9554	0.0826376
18	newly 3 weeks spec	1434.	2232.11	{1571., 2701.}	55.963	0.185679	newly 3 weeks cumul	2751.	3787.43	{2774., 4460.}	38.7848	0.13774	newly 5 weeks spec	4058.	7321.28	{5151., 8810.}	80.416	0.248149	newly 5 weeks cumul	9240.	15 426.	{10 941., 18 166.}	66.9484	0.214873	newly 63 weeks cumul	55 677.	89 637.6	{88 232., 92 631.}	60.9958	0.206237
19	newly 3 weeks spec	1846.	2247.05	{1624., 3088.}	29.4697	0.112044	newly 3 weeks cumul	3207.	3708.05	{2582., 4991.}	24.7412	0.0979912	newly 5 weeks spec	5657.	7726.87	{5662., 10 281.}	40.7826	0.14666	newly 5 weeks cumul	12 216.	15 939.7	{11 975., 21 297.}	35.6619	0.131157	newly 63 weeks cumul	62 777.	76 707.4	{55 305., 92 364.}	28.9563	0.109967
20	newly 3 weeks spec	1317.	6405.14	{987., 14 229.}	398.364	0.53595	newly 3 weeks cumul	2412.	9959.41	{1872., 22 756.}	325.064	0.481414	newly 5 weeks spec	4511.	8128.79	{3152., 13 443.}	97.4935	0.287708	newly 5 weeks cumul	9394.	27 122.9	{7001., 45 877.}	203.436	0.423208	newly 61 weeks cumul	61 521.	64 935.1	{49 638., 92 628.}	27.2387	0.114643
21	newly 3 weeks spec	780.	1318.65	{255., 2038.}	101.329	0.292216	newly 3 weeks cumul	1243.	2027.37	{464., 3065.}	94.3001	0.279217	newly 5 weeks spec	3321.	5172.28	{1063., 9682.}	83.6579	0.294469	newly 5 weeks cumul	6292.	10 086.7	{2400., 17 133.}	89.2853	0.293104	newly 65 weeks cumul	63 478.	62 462.	{50 482., 71 388.}	14.611	0.0662539
22	newly 3 weeks spec	720.	566.02	{70., 1043.}	49.6611	0.452615	newly 3 weeks cumul	1304.	1116.81	{251., 1854.}	45.3474	0.312229	newly 5 weeks spec	2369.	1516.85	{76., 2858.}	51.5935	0.621243	newly 5 weeks cumul	4863.	3569.32	{454., 6368.}	49.3202	0.428647	newly 63 weeks cumul	55 662.	39 192.6	{9551., 89 721.}	61.3683	0.542239
23	newly 3 weeks spec	325.	683.21	{13., 1608.}	183.018	0.64603	newly 3 weeks cumul	484.	948.03	{46., 2144.}	159.87	0.503168	newly 5 weeks spec	3196.	4436.33	{39., 14 082.}	134.152	0.886075	newly 5 weeks cumul	4636.	7510.14	{133., 21 027.}	149.465	0.697941	newly 67 weeks cumul	66 380.	58 906.6	{16 928., 83 827.}	30.4553	0.371123
24	newly 3 weeks spec	356.	4662.51	{0., 15 021.}	1295.54	1.11517	newly 3 weeks cumul	524.	7059.18	{12., 16 842.}	1329.89	0.86627	newly 5 weeks spec	2825.	7244.46	{0., 25 990.}	252.53	1.24685	newly 5 weeks cumul	4283.	19 638.5	{12., 76 437.}	445.809	1.0733	newly 68 weeks cumul	68 010.	60 846.8	{12., 91 541.}	37.0628	0.64743
25	newly 3 weeks spec	331.	1731.24	{0., 1191.}	542.737	1.15065	newly 3 weeks cumul	441.	2180.08	{7., 1473.}	509.619	0.894253	newly 5 weeks spec	2373.	4240.04	{0., 15 356.}	197.982	1.37979	newly 5 weeks cumul	3717.	9291.46	{9., 27 878.}	268.363	1.1537	newly 68 weeks cumul	58 819.	67 436.8	{10., 92 473.}	48.5173	0.703227
26	newly 3 weeks spec	1190.	5351.18	{463., 11 552.}	368.171	0.624952	newly 3 weeks cumul	2010.	7710.67	{1007., 17 245.}	301.045	0.539604	newly 5 weeks spec	4173.	11 460.3	{2481., 17 715.}	192.165	0.507554	newly 5 weeks cumul	8492.	29 270.7	{4865., 43 291.}	260.917	0.557313	newly 62 weeks cumul	69 666.	50 060.4	{36 384., 66 706.}	28.5549	0.183855
27	newly 3 weeks spec	974.	905.86	{600., 1232.}	26.5791	0.120644	newly 3 weeks cumul	1673.	1459.27	{986., 2017.}	25.6874	0.123555	newly 5 weeks spec	3834.	4065.24	{2555., 5515.}	31.1721	0.12255	newly 5 weeks cumul	7556.	7528.69	{4977., 10 343.}	29.0981	0.122156	newly 63 weeks cumul	62 217.	87 356.3	{77 397., 93 522.}	40.4059	0.146068
28	newly 3 weeks spec	2141.	2217.93	{1182., 3497.}	34.6002	0.151177	newly 3 weeks cumul	4227.	4358.56	{2440., 6895.}	30.9189	0.136017	newly 5 weeks spec	4410.	4354.93	{2446., 7649.}	31.919	0.141072	newly 5 weeks cumul	11 858.	12 020.5	{6747., 19 710.}	31.9079	0.140149	newly 62 weeks cumul	54 529.	38 109.3	{32 997., 52 938.}	30.1795	0.160933
29	newly 3 weeks spec	1545.	1994.17	{1252., 2864.}	38.2168	0.136091	newly 3 weeks cumul	2666.	3369.84	{2191., 4728.}	35.4291	0.12841	newly 5 weeks spec	5090.	6504.21	{4316., 9120.}	36.544	0.131157	newly 5 weeks cumul	10 609.	13 755.9	{9095., 18 920.}	37.0347	0.131391	newly 63 weeks cumul	65 892.	63 420.3	{37 795., 89 115.}	21.4886	0.0980253
30	newly 3 weeks spec	1626.	1427.99	{1328., 1543.}	12.1777	0.0571247	newly 3 weeks cumul	3106.	2771.57	{2584., 2977.}	10.7672	0.0500842	newly 5 weeks spec	3975.	3557.43	{3305., 3822.}	10.5049	0.0489577	newly 5 weeks cumul	9727.	8638.62	{8045., 9269.}	11.1893	0.0521901	newly 59 weeks cumul	54 604.	85 660.4	{86 217., 86 803.}	56.8757	0.195161
31	newly 3 weeks spec	312.	608.78	{1., 277.}	249.372	1.30709	newly 3 weeks cumul	454.	858.94	{6., 335.}	242.863	1.08341	newly 5 weeks spec	3043.	1631.79	{0., 3388.}	111.475	1.74218	newly 5 weeks cumul	4393.	3288.19	{8., 5540.}	130.66	1.44334	newly 68 weeks cumul	60 937.	66 304.5	{9., 93 163.}	42.4934	0.628273
32	newly 3 weeks spec	434.	1392.1	{338., 2689.}	231.631	0.42887	newly 3 weeks cumul	710.	1814.51	{480., 3498.}	169.052	0.359125	newly 5 weeks spec	2775.	10 583.8	{2822., 20 285.}	286.928	0.52024	newly 5 weeks cumul	4597.	17 105.	{4416., 33 167.}	278.237	0.496034	newly 66 weeks cumul	62 201.	87 259.8	{77 944., 93 800.}	41.2955	0.149237
33	newly 3 weeks spec	975.	7143.48	{2628., 12 869.}	632.665	0.772777	newly 3 weeks cumul	1516.	9257.35	{3354., 16 868.}	510.932	0.696677	newly 5 weeks spec	5236.	21 283.1	{16 024., 25 202.}	306.477	0.59908	newly 5 weeks cumul	8992.	47 165.7	{29 804., 66 030.}	424.529	0.698283	newly 66 weeks cumul	68 723.	86 547.5	{75 872., 93 006.}	27.7344	0.106074
34	newly 3 weeks spec	1779.	945.7	{774., 1133.}	46.8409	0.27948	newly 3 weeks cumul	3127.	1890.98	{1579., 2196.}	39.5273	0.222346	newly 5 weeks spec	4645.	2262.82	{1813., 2686.}	51.2848	0.318256	newly 5 weeks cumul	10 816.	5642.63	{4636., 6648.}	47.8307	0.287458	newly 58 weeks cumul	64 121.	67 416.6	{54 389., 72 377.}	9.23633	0.0395214
35	newly 3 weeks spec	295.	148.44	{1., 293.}	90.3119	0.995717	newly 3 weeks cumul	455.	210.84	{13., 417.}	83.833	0.800195	newly 5 weeks spec	2408.	1157.94	{0., 2372.}	99.7633	1.30189	newly 5 weeks cumul	3628.	1843.24	{20., 3439.}	98.8252	1.05938	newly 67 weeks cumul	61 775.	69 613.4	{26., 92 028.}	41.6914	0.558282
36	newly 3 weeks spec	489.	279.02	{104., 475.}	52.638	0.416594	newly 3 weeks cumul	783.	444.57	{180., 731.}	51.1022	0.368506	newly 5 weeks spec	2506.	1602.37	{456., 2877.}	56.1425	0.445917	newly 5 weeks cumul	4350.	2730.43	{808., 4858.}	54.4393	0.400269	newly 65 weeks cumul	66 421.	83 636.3	{77 463., 91 166.}	32.377	0.19706
37	newly 3 weeks spec	391.	10 973.9	{1884., 26 543.}	2708.63	1.32831	newly 3 weeks cumul	633.	15 729.8	{2402., 36 859.}	2386.96	1.25423	newly 5 weeks spec	3280.	11 888.8	{6653., 15 502.}	266.347	0.596957	newly 5 weeks cumul	5202.	42 201.6	{22 417., 66 590.}	713.257	0.911257	newly 67 weeks cumul	56 370.	54 264.8	{49 700., 68 947.}	14.2894	0.104844
38	newly 3 weeks spec	728.	1119.91	{322., 2142.}	84.4025	0.351223	newly 3 weeks cumul	1154.	1642.44	{441., 2987.}	72.669	0.299024	newly 5 weeks spec	4350.	6593.57	{1020., 12 545.}	88.9074	0.412119	newly 5 weeks cumul	7337.	11 215.4	{2228., 21 238.}	87.9283	0.364509	newly 65 weeks cumul	69 743.	78 346.8	{38 595., 93 489.}	27.6036	0.185738
39	newly 3 weeks spec	1971.	2166.32	{999., 3405.}	38.8016	0.180976	newly 3 weeks cumul	3791.	4203.11	{1859., 6352.}	37.9312	0.17563	newly 5 weeks spec	4327.	4388.28	{2368., 6035.}	27.2665	0.134266	newly 5 weeks cumul	11 151.	11 884.	{6342., 17 377.}	32.6339	0.154385	newly 62 weeks cumul	53 564.	35 972.6	{34 231., 37 336.}	32.8419	0.173196
40	newly 3 weeks spec	1025.	4445.05	{430., 14 064.}	357.738	0.580009	newly 3 weeks cumul	1775.	7077.87	{928., 22 406.}	321.542	0.516822	newly 5 weeks spec	3528.	6513.55	{1459., 12 637.}	112.412	0.414116	newly 5 weeks cumul	7401.	20 012.8	{3076., 45 651.}	196.334	0.469024	newly 65 weeks cumul	51 673.	43 168.6	{35 362., 55 764.}	20.4878	0.162649
41	newly 3 weeks spec	676.	34 355.6	{16 884., 44 738.}	4982.19	1.67551	newly 3 weeks cumul	1025.	49 110.5	{21 814., 70 206.}	4691.26	1.6356	newly 5 weeks spec	4452.	9722.53	{1735., 17 047.}	139.759	0.381626	newly 5 weeks cumul	7242.	83 110.8	{75 226., 91 092.}	1047.62	1.05762	newly 67 weeks cumul	64 974.	87 695.5	{78 231., 93 925.}	34.9701	0.128532
42	newly 3 weeks spec	1593.	1404.84	{1146., 1567.}	12.3139	0.0595717	newly 3 weeks cumul	2901.	2638.03	{2229., 2945.}	10.072	0.0477286	newly 5 weeks spec	4138.	3782.52	{3088., 4256.}	10.2523	0.0491984	newly 5 weeks cumul	9608.	8784.63	{7184., 9780.}	9.94432	0.0473907	newly 60 weeks cumul	61 993.	84 320.2	{71 762., 91 500.}	37.2082	0.136337
43	newly 3 weeks spec	1692.	1931.04	{1610., 2183.}	18.1584	0.0733522	newly 3 weeks cumul	3552.	3914.27	{3370., 4332.}	13.8775	0.0571418	newly 5 weeks spec	3812.	4117.09	{3206., 4704.}	15.1047	0.0637896	newly 5 weeks cumul	9984.	10 945.9	{8792., 12 277.}	14.852	0.0619034	newly 59 weeks cumul	48 969.	66 371.9	{39 502., 81 782.}	43.7602	0.153543
44	newly 3 weeks spec	459.	459.92	{91., 860.}	56.5098	0.339414	newly 3 weeks cumul	732.	677.87	{175., 1202.}	50.0997	0.295076	newly 5 weeks spec	2554.	3242.67	{537., 6849.}	76.3316	0.42339	newly 5 weeks cumul	4392.	5209.33	{889., 10 877.}	69.2452	0.358123	newly 66 weeks cumul	67 035.	77 280.7	{57 180., 92 434.}	26.6065	0.199749
45	newly 3 weeks spec	956.	928.95	{76., 1853.}	62.0136	0.463512	newly 3 weeks cumul	1721.	1687.33	{186., 3395.}	59.0848	0.377683	newly 5 weeks spec	3513.	2633.81	{126., 5211.}	54.3527	0.542438	newly 5 weeks cumul	7079.	6002.67	{536., 12 016.}	56.8573	0.437383	newly 64 weeks cumul	64 288.	34 125.2	{12 837., 38 876.}	46.9183	0.395743
46	newly 3 weeks spec	720.	957.03	{56., 1565.}	86.2542	0.459519	newly 3 weeks cumul	1225.	1488.63	{118., 2393.}	74.4955	0.374607	newly 5 weeks spec	3211.	3781.82	{182., 6535.}	75.0501	0.51487	newly 5 weeks cumul	5975.	7369.45	{494., 12 139.}	78.4474	0.439927	newly 63 weeks cumul	66 403.	47 491.9	{34 481., 60 254.}	29.0633	0.309027
47	newly 3 weeks spec	2512.	3282.29	{2275., 4149.}	36.0729	0.130976	newly 3 weeks cumul	5499.	6716.55	{4591., 8284.}	28.9945	0.110259	newly 5 weeks spec	4726.	5679.2	{3925., 7154.}	29.1976	0.111082	newly 5 weeks cumul	13 753.	16 963.3	{11 662., 20 182.}	30.0906	0.113406	newly 59 weeks cumul	63 771.	55 321.8	{32 444., 87 093.}	42.4286	0.216763
48	newly 3 weeks spec	1312.	2025.91	{766., 3097.}	71.2691	0.240025	newly 3 weeks cumul	2348.	3388.33	{1497., 5116.}	60.9042	0.214545	newly 5 weeks spec	4095.	6521.16	{2526., 9915.}	75.3304	0.256324	newly 5 weeks cumul	8888.	13 848.8	{5810., 20 457.}	71.7966	0.244248	newly 64 weeks cumul	66 105.	55 707.6	{37 603., 70 372.}	17.7772	0.0897138
49	newly 3 weeks spec	197.	4149.76	{0., 14 067.}	2071.02	1.13702	newly 3 weeks cumul	276.	5173.59	{10., 15 793.}	1832.37	0.868675	newly 5 weeks spec	2370.	8326.51	{0., 24 847.}	333.154	1.30255	newly 5 weeks cumul	3259.	21 072.1	{10., 75 233.}	622.706	1.09193	newly 68 weeks cumul	66 569.	61 738.7	{10., 92 323.}	38.5875	0.675601
50	newly 3 weeks spec	580.	6138.57	{360., 14 929.}	971.743	0.804473	newly 3 weeks cumul	912.	7953.71	{585., 18 834.}	785.938	0.734207	newly 5 weeks spec	4132.	15 975.8	{2399., 25 939.}	302.453	0.602703	newly 5 weeks cumul	6745.	36 352.1	{4326., 65 953.}	453.978	0.699012	newly 67 weeks cumul	62 718.	75 832.9	{55 027., 91 239.}	27.6535	0.108841
